# Non-invasive Fetal Electrocardiography for Intrapartum Cardiotocography

**DOI:** 10.3389/fped.2020.599049

**Published:** 2020-12-09

**Authors:** Rik Vullings, Judith O. E. H. van Laar

**Affiliations:** ^1^Biomedical Diagnostics Lab Eindhoven, Department of Electrical Engineering, Eindhoven University of Technology, Eindhoven, Netherlands; ^2^Nemo Healthcare, Veldhoven, Netherlands; ^3^Máxima Medical Center, Veldhoven, Netherlands

**Keywords:** electrophysiology, cardiotocography, fetal heart rate, fetal electrocardiogram, signal processing, artificial intelligence

## Abstract

Fetal monitoring is important to diagnose complications that can occur during pregnancy. If detected timely, these complications might be resolved before they lead to irreversible damage. Current fetal monitoring mainly relies on cardiotocography, the simultaneous registration of fetal heart rate and uterine activity. Unfortunately, the technology to obtain the cardiotocogram has limitations. In current clinical practice the fetal heart rate is obtained via either an invasive scalp electrode, that poses risks and can only be applied during labor and after rupture of the fetal membranes, or via non-invasive Doppler ultrasound technology that is inaccurate and suffers from loss of signal, in particular in women with high body mass, during motion, or in preterm pregnancies. In this study, transabdominal electrophysiological measurements are exploited to provide fetal heart rate non-invasively and in a more reliable manner than Doppler ultrasound. The performance of the fetal heart rate detection is determined by comparing the fetal heart rate to that obtained with an invasive scalp electrode during intrapartum monitoring. The performance is gauged by comparing it to performances mentioned in literature on Doppler ultrasound and on two commercially-available devices that are also based on transabdominal fetal electrocardiography.

## 1. Introduction

One in every five pregnant women experiences complications during her pregnancy (1). Although most of these complications are relatively harmless, some are more severe and will lead to fetal morbidity, or even mortality. The most important pregnancy complications, in terms of severity and occurrence, are premature birth, birth hypoxia, intrauterine growth restriction, and congenital anomalies. Together, this “big four” of pregnancy complications accounts for the majority of perinatal morbidities and mortalities (2).

Early detection of these pregnancy complications is of the utmost importance to prevent irreversible damage, but is unfortunately hampered by limitations of the technology that is used in daily clinical practice. Essentially, this technology comprises of cardiotocography and ultrasound imaging. The former constitutes a simultaneous registration of fetal heartrate (FHR) and maternal uterine activity (UA). It is used to screen for patterns in FHR or heartrate variability that could reveal a compromised condition, e.g., acidaemia (3). The latter is mostly used to screen for anomalies such as growth restriction or congenital heart disease (CHD).

The cardiotocogram (CTG) is obtained in daily practice by either invasive means, using a fetal scalp electrode (FSE) and intrauterine pressure catheter (IUPC), or non-invasive means, using a Doppler ultrasound probe and an external tocodynamometer. The invasive methods suffer from the limitations that they can only be used during labor after rupture of the fetal membranes and impose risks to mother and child (4). In some countries, these invasive methods are therefore no longer used. The non-invasive methods can be used throughout pregnancy, but are known to be unreliable (5, 6).

The Doppler ultrasound employed in cardiotocography consists of a rather narrow beam of ultrasound that insonifies a small volume in the maternal abdomen (7). If the fetal heart is within this volume, the signal to noise ratio of the reflected ultrasound beam is typically good enough to extract a reliable FHR. However, movement of mother or fetus, or high body mass of mother causes poor insonification of the fetal heart, with corresponding poor reliability of the derived FHR (8, 9). Also in preterm fetuses or multiple pregnancies, Doppler ultrasound is known to perform poorly.

Over the past decades, extensive research has focused on non-invasive fetal electrophysiological recordings for measurement of the CTG (10). Other than Doppler ultrasound, these electrophysiological recordings are hardly affected by movement and body mass (11, 12). However, the recordings are corrupted by many electrical interferences, of which the maternal heart is the dominant source. Many studies have been published on methods to remove this interference, i.e., the maternal electrocardiogram (ECG), and virtually all with good performance (13–18). Yet, in many practical situations, removal of the maternal ECG alone is not enough to enable reliable measurement of the FHR (18, 19). For example, during labor the maternal abdominal muscles cause interferences that exceed the fetal electrical cardiac activity (i.e., fetal ECG) in terms of amplitude and that overlap in the frequency domain.

Perhaps due to these practical limitations, to date, only a few solutions exist that constitute an electrophysiology-based device for CTG acquisition and that are ready for use in clinical practice. A few examples of such solutions include the GE Novii (GE, USA, formerly the Monica Healthcare Novii), the Philips Avalon Beltless (Philips, the Netherlands), and the Nemo Fetal Monitoring System (Nemo Healthcare, the Netherlands, of which one of the authors is co-founder). The use of these solutions in clinical practice is still fairly limited, mainly due to the relatively poor performance during second stage of labor (6, 12).

This paper proposes a practical solution to solve the problems that limit the application of non-invasive fetal electrocardiography-based cardiotocography, especially during second stage of labor. The focus of the paper lies on the acquisition of FHR; for assessing the maternal uterine activity, the reader is referred to literature such as (20–22). The performance of the method is assessed by comparing the FHR to that determined with a simultaneously applied FSE during intrapartum monitoring.

This paper is organized as follows: In section 2, the methodology for acquiring electrophysiological data and the signal processing toward cardiotocography are discussed and details are provided on the datasets used in this paper. In section 3, results of the signal processing methods are illustrated and in section 4 the results are discussed.

## 2. Materials and Methods

**Figure 1:**
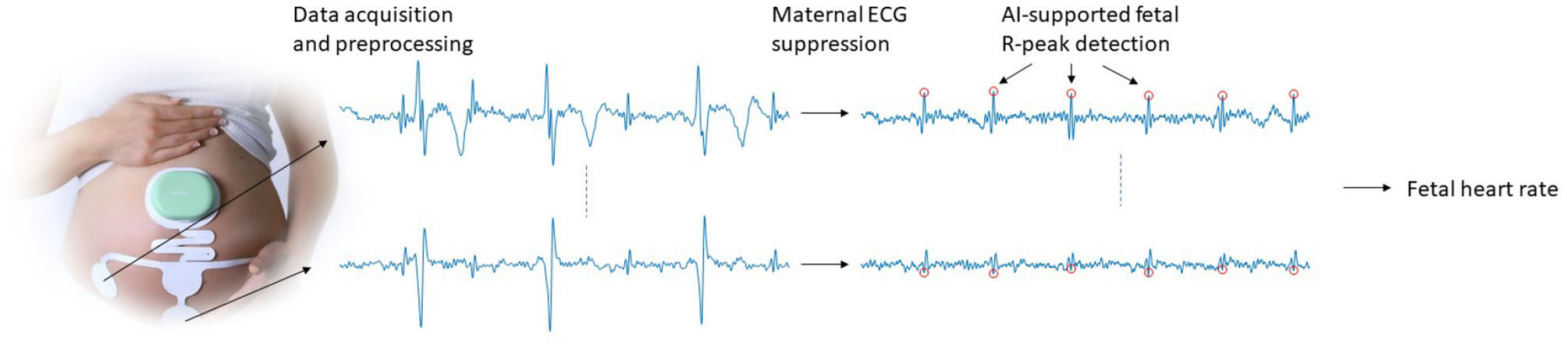
Schematic illustration of the data acquisition and signal processing steps that are used to obtain FHR.

The various methodological steps that are needed for acquiring electrophysiological data and signal processing toward FHR are schematically depicted in Figure 1. These steps will be discussed in more detail in the subsections below. Because some of these steps have been described in detail in other publications, we will discuss the methodology in terms of a “cookbook recipe” and will focus our description on the steps that have not yet been published in detail, i.e., the FHR detection step with artificial intelligence (AI) extension.

### 2.1. Data

The study protocol for the data used in this study was approved by the institutional review board of the Máxima Medical Center in December 2017 (NL63732.015.17). Women in established labor, carrying a healthy singleton fetus in cephalic presentation and with a gestational age between 36 and 42 weeks were eligible to participate. After written informed consents, participants received an adhesive electrode patch (Nemo Healthcare BV, the Netherlands) that comprises four unipolar electrodes, a ground electrode, and a common reference. Data were recorded locally on the patch and digitized at 500 Hz sample rate with a resolution of 22 nV. Subsequently, data was transmitted wirelessly to a data processing device to yield instantaneous output of FHR, maternal heart rate, and UA. In parallel, digitized signals were stored on the data acquisition system to enable offline processing. All results in this paper are obtained via offline processing of stored data to allow for quantitative evaluation. To facilitate such evaluation, for all patients, a simultaneous FHR recording using a FSE was performed. The FHR of the scalp electrode was determined by a Philips Avalon FM30 cardiotocograph (Philips, the Netherlands) at sampling period of 4 Hz. The output of the cardiotocograph was stored digitally as well.

In total, 136 recordings were performed with an average duration of 185 ± 135 min, with the shortest recording 17 min and the longest 600 min. The evaluation of the presented method was only performed on 26 of these 136 recordings. The other 110 measurements were used to develop the methods presented in this study. More details on this splitting of the dataset are provided in section 2.2.3.2. Details on the age and body-mass-index of the mother are provided in Table 1; other relevant details such as fetal gender, weight, and presentation were unfortunately not registered.

**Table 1:** Age and body-mass-index (BMI) of the patients included in the study, subdivided over patients that were used in the training of the proposed methods and patients that were used in the evaluation of the methods.

	***n***	**Age (years)**	**BMI (kg·m^−2^)**	***p*-value**
Training	110^*^	31.2 ± 4.4	28.5 ± 5.0	0.74
Testing	26^†^	30.9 ± 2.3	28.1 ± 5.5	0.70

*The number of patients per group is indicated by n. A unpaired t-test was performed to determine whether the age and BMI between train and test datasets were significantly different*.

* *For two subjects from the train dataset the age was not known, for one subject the BMI was not known*.

† *For one subject from the test dataset the BMI was not known*.

### 2.2. Signal Processing

#### 2.2.1. Preprocessing

The recorded signals were preprocessed to suppress interferences from e.g., abdominal muscles, baseline wander, and mains powerline. This preprocessing consists of the application of zero-phase delay highpass and lowpass filters, with cutoff frequencies of 1 and 70 Hz, respectively.

For the mains powerline, depending on geographical location (e.g., 50 Hz for Europe, 60 Hz for USA), a Kalman smoother was used to effectively suppress the powerline interference, while avoiding so-called ringing that characterizes conventional (in)finite impulse response filters (23). Details of the applied Kalman smoother are provided in (23).

#### 2.2.2. Maternal ECG Suppression

After preprocessing, the dominant interference in the electrophysiological abdominal recordings is the maternal ECG. As mentioned in section 1, many studies have been published on methods for suppressing the maternal ECG. Most of these methods perform good enough to the point where the (possible) residuals of maternal ECG are no longer the dominant interference and where the methods do not cause any significant degradation to the quality of the remaining fetal ECG.

In this work, we use a template-based maternal ECG suppression method. First maternal QRS complexes are detected using a low-complexity R-peak detection method, presented in (24). Then the recorded signals are segmented, based on the detected maternal R-peaks, to yield one maternal ECG complex per segment. Each ECG complex is then further segmented to yield individual ECG waves. For each wave, a template is generated from the linear prediction of corresponding waves from preceding ECG complexes. The wave templates are subsequently combined to yield a template ECG. This method is discussed in detail in (15). Because the FHR is typically not correlated to the maternal heart rate, fetal ECG complexes occur in random places in the maternal ECG segments. In the linear prediction step, these fetal ECG complexes are therefore strongly attenuated in the template.

**Figure 2:**
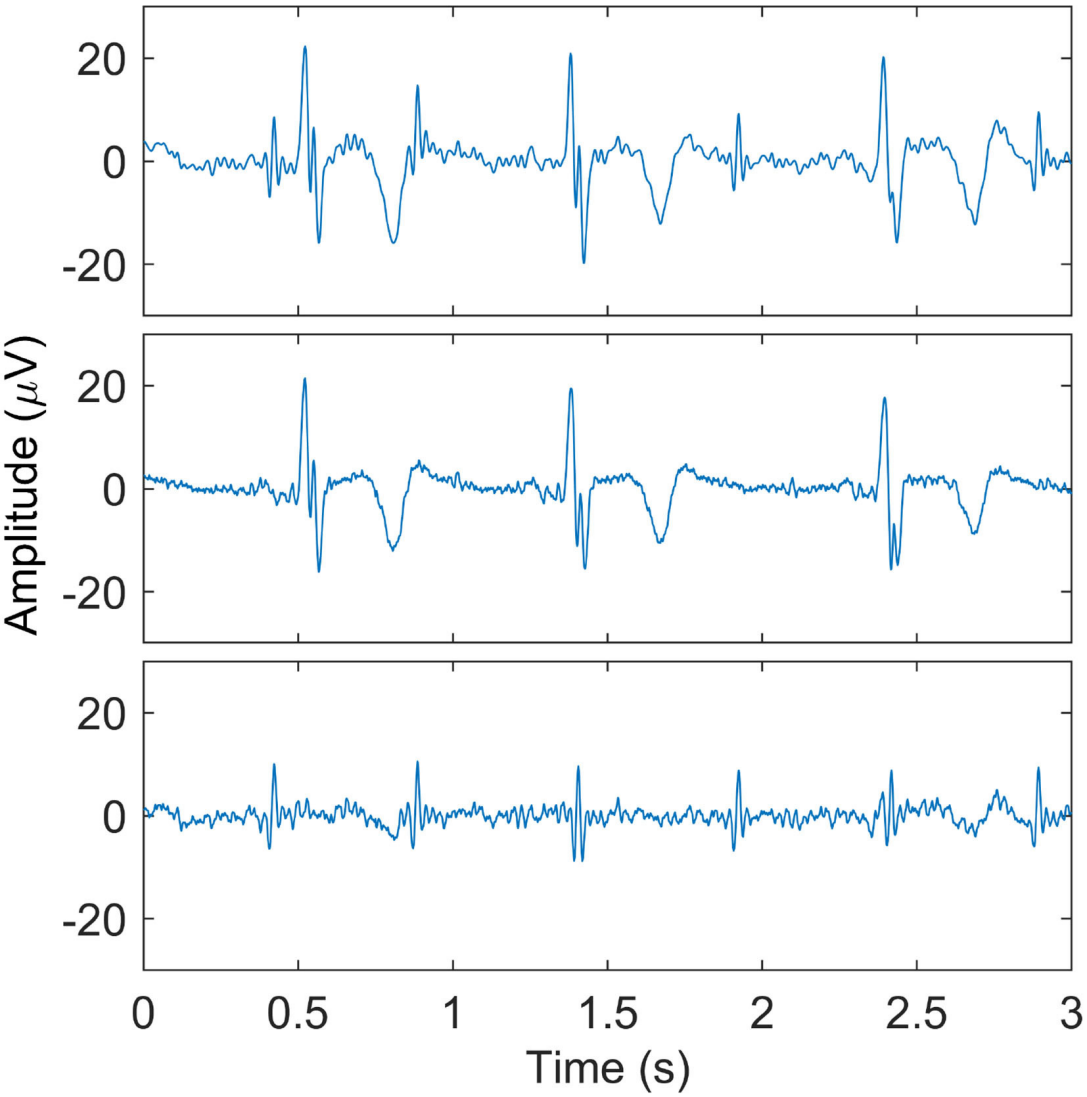
Illustration of maternal ECG estimation. In the **(top)** panel, one of the recorded signals after preprocessing is shown. The **(middle)** panel shows the estimate of the maternal ECG signal, and the **(bottom)** panel shows the signal that results after subtracting the maternal ECG estimate. Here, the fetal QRS complexes are clearly visible.

As a final step in the maternal ECG suppression, the templates per ECG complex are concatenated to produce an estimate of the maternal ECG signal which is then subtracted from the recorded signal, ideally preserving the fetal ECG. This procedure is illustrated in Figure 2.

#### 2.2.3. Fetal Heart Rate Detection

**Figure 3:**
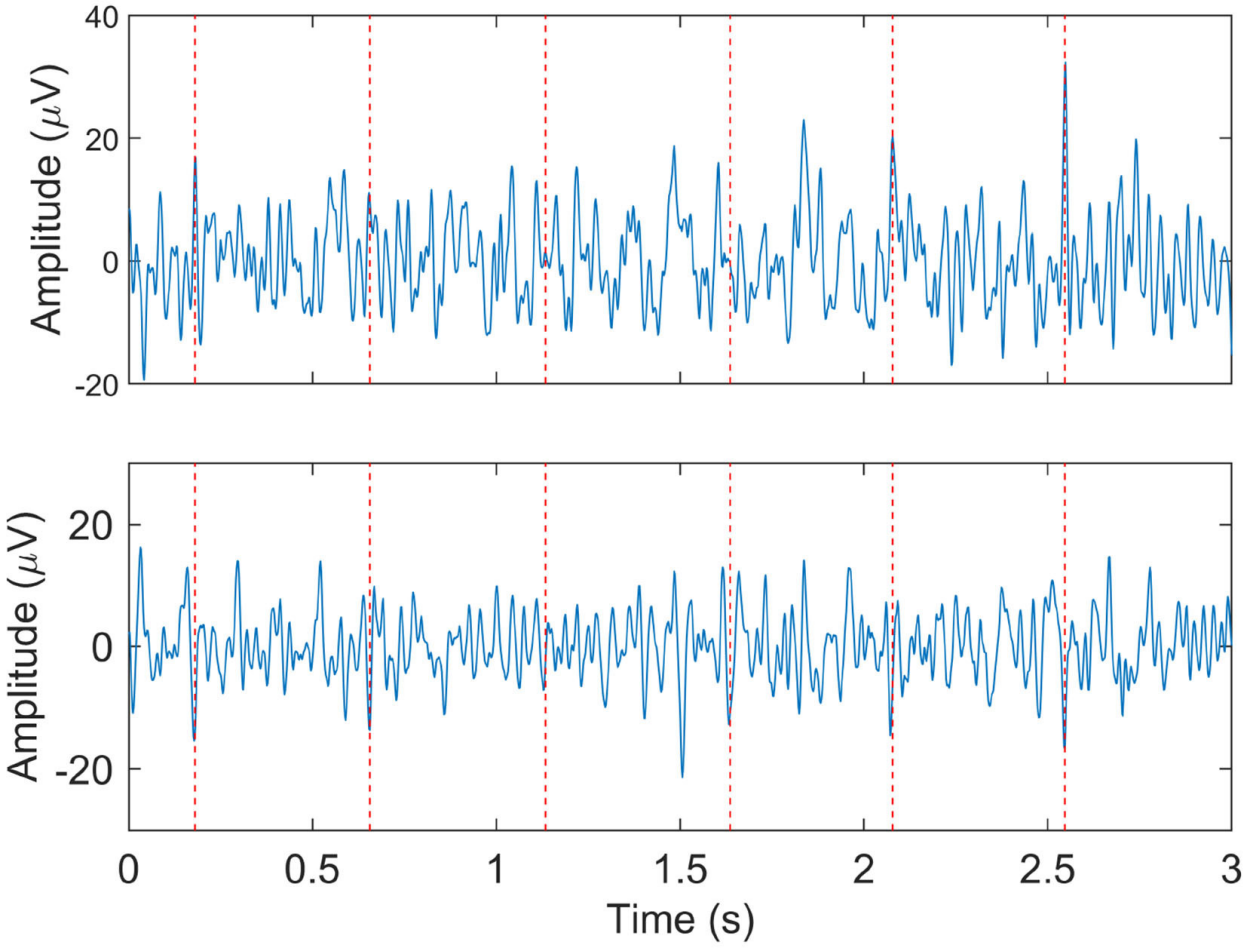
Example of relatively low quality fetal ECG signals after maternal ECG suppression. The two panels show the signals from two different electrodes at the same moment. The dashed vertical lines indicate the locations at which the fetal heart rate detection method did detect fetal R-peaks.

Despite the accurate maternal ECG suppression that can be achieved, often the fetal ECG is still obscured by other interferences that remain after maternal ECG cancelation. In such cases, reliable detection of the FHR is still challenging. In Figure 3 and example of a low-quality fetal ECG signal is shown.

##### 2.2.3.1. Hierarchical Probabilistic Framework for R-peak Detection

In (19), we have introduced a model-based approach for detecting the fetal R-peaks. This approach leverages models on the fetal QRS waveform, on the heartrate, and on the noise dynamics to yield a robust fetal R-peak detection, even in case of low-quality signals. In this work, we extend our previous method with AI to further improve its robustness.

The method by Warmerdam et al. is based on the following state-space equation:

(1) $$\begin{array}{l} \mu_{k+1}=\mu_{k}+{\vec{w}}_{k}{\vec{\theta }}_{k+1}+v_{k+1} \end{array}$$

(2) $$\begin{array}{l} {\vec{y}}_{k+1}=G\left(\vec{t},\mu_{k+1},{\vec{z}}_{k+1}\right)+{\vec{\xi }}_{k+1}. \end{array}$$

In Equation (1), μ_*k*+1_ is the location of the (*k* + 1)^th^ fetal R-peak, ${\vec{w}}_{k}$ are previously detected interbeat (i.e., RR) intervals, ${\vec{\theta }}_{k+1}$ are the coefficients from an autoregressive (AR) model, and *v*_*k*+1_ is a term that accounts for heartrate variability. Essentially, the location of the next fetal R-peak is estimated to be the location of the previous R-peak, plus the expected RR-interval, plus a random term. This random term *v*_*k*+1_ is sampled from a zero-mean normal distribution.

In Equation (2), ${\vec{y}}_{k+1}$ is the (*k* + 1)^th^ segment of the recorded signal *y*, *G*(·) is a function that describes the linear combination of three Gaussian functions:

(3) $$\begin{array}{l} G\left(t,\mu_{k},\vec{z}\right)=\left(a_{1}+a_{2}\left(t-\mu_{k}\right)+a_{3}\left(1-\frac{{\left(t-\mu_{k}\right)}^{2}}{b^{2}}\right)\right)e^{-\frac{{\left(t-\mu_{k}\right)}^{2}}{2b^{2}}}, \end{array}$$

${\vec{z}}_{k+1}=\left[a_{1},a_{2},a_{3},b\right]$ are the parameters (i.e., amplitudes $\vec{a}$ and variance *b*) for these Gaussian functions, and ${\vec{\xi }}_{k+1}$ represents measurement noise.

Using Bayes' rule, the *maximum a posteriori* estimate ${\hat{\mu }}_{k+1}$ can be obtained as:

(4) $$\begin{array}{l} {\hat{\mu }}_{k+1}=\arg\underset{\mu }{\max}\{-\frac{{\left(\mu_{k+1}-{\hat{\mu }}_{k}+{\hat{w}}_{k+1}^{T}{\vec{\theta }}_{k+1}\right)}^{2}}{\Gamma^{\text{HR}}}\\ \;-{\left({\vec{y}}_{k+1}-{\hat{\vec{y}}}_{k+1}\right)}^{T}\Gamma^{{\text{QRS}}^{-1}}\left({\vec{y}}_{k+1}-{\hat{\vec{y}}}_{k+1}\right)\}. \end{array}$$

Here, Γ^HR^ and Γ^QRS^ are estimates of the heart rate variability and measurement noise, respectively, and ${\hat{\vec{y}}}_{k+1}$ is the estimate of the recorded signal (cf. Equation 2). The first term on the righthandside is in following paragraphs referred to as the *prior* model and the second term the *likelihood* model. All model parameters are updated using (extended) Kalman filters. For exact details on this method, the reader is referred to (19).

##### 2.2.3.2. Artificial Intelligence Extension

Although the method by Warmerdam et al. was designed and shown to be robust against low-quality signals, situations occur where its performance rapidly decreases. This happens for instance when noise or interferences cause erroneous updates of the model parameters. At that point, a vicious circle will cause the next R-peak detection to go wrong, which in turn further diverges the model parameters, and so on. Therefore, in this paper we propose an AI-based extension of the method to prevent such scenarios.

In this AI-based extension the RR-intervals are estimated using an AI model that is described in (25). Although this model at first seems to outperform other methods, its main limitation is that it can provide FHR outputs that look physiologically plausible but are in fact incorrect (25). Yet, in this work we use the RR-intervals detected by the AI model to validate the RR-interval estimate by the AR model: ${ŵ}_{k+1}^{T}{\vec{\theta }}_{k+1}$. As described above, heartrate variability is modeled in the state-space representation as random values *v*_*k*+1_ sampled from a zero-mean normal distribution. Assuming that this distribution has variance Σ_*k*+1_, in case of poor agreement between the AR and AI model, this variance is increased as:

(5) $$\begin{array}{l} \Sigma_{k+1}\leftarrow \Sigma_{k+1}+\left|{ŵ}_{k+1}^{T}{\vec{\theta }}_{k+1}-RR_{{\text{AI}}_{k+1}}\right|, \end{array}$$

where *RR*_AI_ is the RR-interval determined by the AI model. In case the two models are not in agreement, the variance of the prior model in Equation (4) is increased to the degree where this distribution can become virtually flat and no prior knowledge on the location of the new fetal R-peak is assumed. New fetal R-peak detections will therefore only be based on the agreement between the expected shape ${\hat{\vec{y}}}_{k+1}$ of the fetal R-peak and the recorded signal ${\vec{y}}_{k+1}$.

To prevent erroneous updates of the model parameters, when the difference between the RR-intervals detected by the presented method and the AI model exceeds 0.05 s, the model parameters are not updated. Likewise, no FHR output is shown to the clinician to prevent showing unreliable FHR information.

To train the AI model, 110 of the 136 recordings that had simultaneous FHR recording with the presented method as well as with FSE were randomly selected. The remaining 26 recordings were used as holdout set to evaluate the performance. A validation of only 26 recordings is relatively small, albeit that these 26 recordings together comprise of 84.2 h of multi-channel abdominal fetal electrophysiological recordings. With common use of such AI, a significant risk of overfitting to the training data might occur. In the method proposed here, this risk is largely mitigated by using the RR-intervals that are determined by the AI to increase the variance Σ_*k*+1_. In case the RR-intervals would be overfitted, this variance would increase and the fetal R-peak will be based more on the *likelihood* model of Equation (4). Yet, to provide insights in the potential overfitting of the AI to the training data, in the section 3 we will provide results from the validation data as well as from the training data.

##### 2.2.3.3. Postprocessing of Fetal Heart Rates

The FHR can be calculated from the detected fetal R-peak positions, yielding a FHR on a beat-to-beat basis. However, to facilitate the communication with central monitoring systems (CMS) and ease the comparison to other methods for which the FHR has been acquired via CMS, this beat-to-beat FHR was resampled to 4 Hz using linear interpolation. Prior to resampling, outliers, which were defined as FHR values that differ more than 20 % from the previous FHR values (26), were omitted and replaced by zeros.

### 2.3. Methodology for Evaluation

For evaluation of the presented method, the performance of FHR detection can be assessed by comparing the FHR to that of the FSE. Moreover, the performance of our method can be gauged by comparing it to that of other methods reported in literature. However, the various models in our method are initialized such that they work optimally when always the same electrode positions are chosen. This is also illustrated in Figure 1, where it is shown that a single electrode patch is used to guarantee consistent placement of the electrodes. Because of this limitation, we cannot apply our method to public datasets such as the Physionet Non-invasive Fetal ECG Database, as in this dataset “electrode positioning was varied in order to improve SNR” (27). Yet, we can compare our method to results from (6) and (12) where similar devices are tested on similar datasets. Both devices from these studies (i.e., Monica Healthcare AN24 and Nemo Fetal Monitoring System) use transabdominal electrodes to record a multi-channel fetal ECG and use proprietary signal processing methods to extract the FHR from these recordings. In fact, with respect to the study of Lempersz et al., our work presents an extension of the algorithms and datasets presented in that paper. With respect to the study of Cohen et al., it should be noted that the comparison on FHR detection performance is only indirect because different datasets are used.

As evaluation metrics, we opt to express the performance in FHR estimation in terms of *success rate, reliability*, and *accuracy*. Here, *success rate* is defined as the percentage of time the method can provide a FHR estimation (6, 12). *Reliability* is expressed in terms of *positive percent agreement (PPA)* which is defined as the percentage of FHR values provided by the method that are within a 10% margin from a valid simultaneous FHR from the FSE (6). For *accuracy* we use bootstrapping of the absolute differences between the FHR from our method and that of the FSE. This metric is different from the definitions by Cohen et al. and by Lempersz et al., which are also different from each other. The reason for choosing a different way of calculating the *accuracy* is described below.

In Cohen et al., the *accuracy* is determined by the root-mean-squared error of the difference between the FHR from two devices vs. the expected difference that is determined by regression in the Bland-Altman plot. In case of a bias between the two FHR measurements, the regression in the Bland-Altman plot will correct for this, yielding a very small metric, even when the FHR determined from the non-invasive measurements differs significantly from the FHR from the FSE. In their paper, Cohen et al. did present the slope and y-intercept of the regression plots, making it possible to appropriately appreciate their findings, but in this work we prefer to show the *accuracy* as a metric that can be interpreted independent from other metrics such as the slope of the regression.

In Lempersz et al. the *accuracy* is determined based on bootstrapping over differences between the FHR from the non-invasive measurements and that of the FSE. If the FHR from the non-invasive measurements would be inaccurate, but without a significant bias, again the metric would be very small. By using the absolute difference instead of the signed differences, this issue is resolved.

Next to mean and standard deviations of the *success rate, reliability*, and *accuracy*, we also provide 95% confidence intervals (CI) and for the *accuracy* limits of agreements. For *accuracy*, all analyses are done using bootstrapping (28). Each bootstrap sample was generated by drawing a random pair of non-invasive FHR and FSE FHR for each woman included in the analysis. For the bootstrap sample, mean absolute difference and standard deviation of absolute differences were determined. This process was repeated 10,000 times to yield a large distribution for the mean absolute difference. The average accuracy and 95% CI were determined by taking the mean of the distribution and the 2.5 and 97.5% centile of the distribution, respectively. From the 10,000 bootstrap samples, also the mean standard deviation was determined, which was subsequently used to calculate limits of agreement as mean accuracy ± 2 × mean standard deviation (12).

## 3. Results

**Figure 4:**
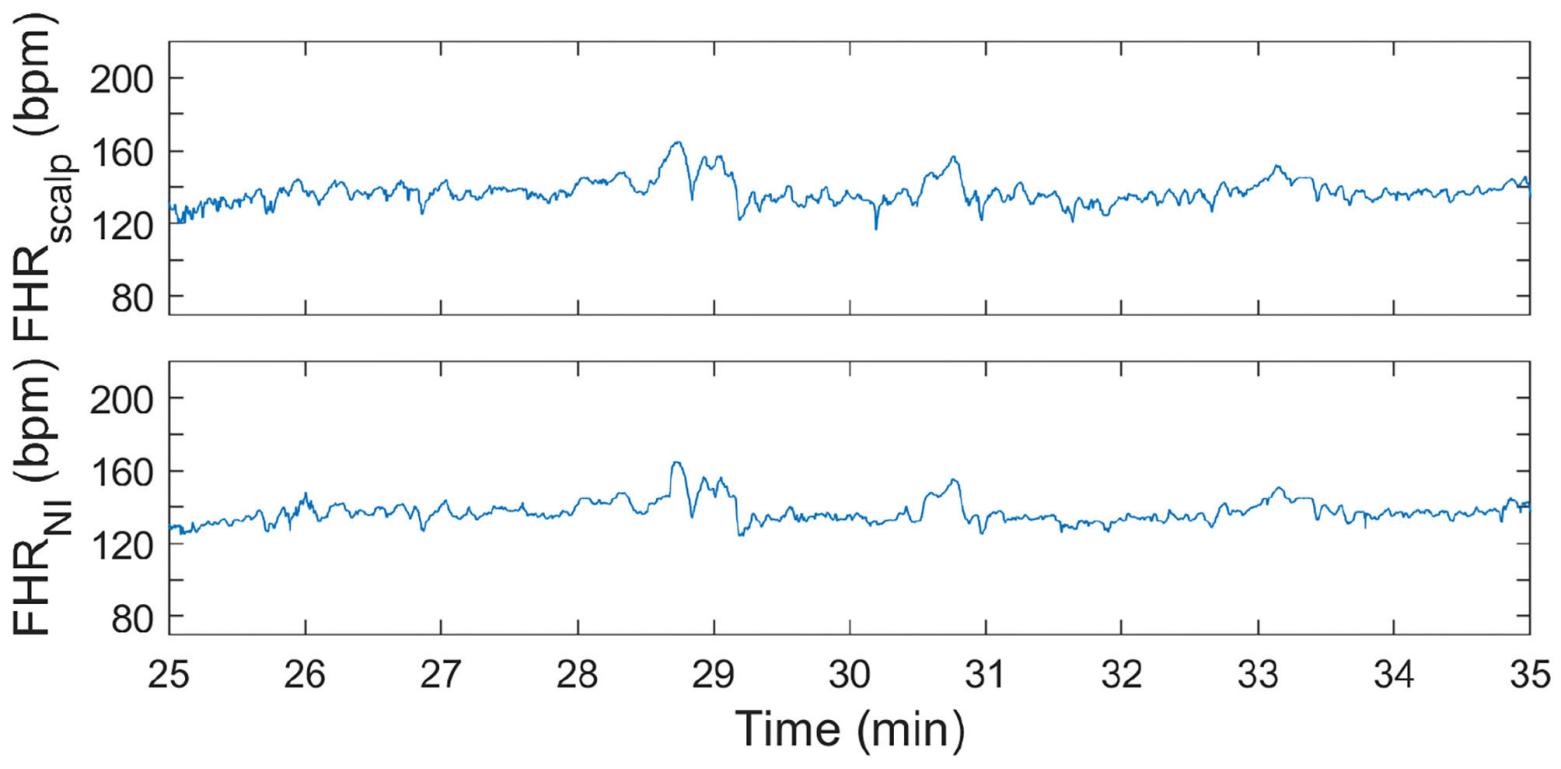
FHR tracing during first stage of labor. In the **(top)** panel, the FHR from FSE is depicted. In the **(bottom)** panel, the FHR determined from the non-invasive fetal ECG (FHR_NI_) with the proposed methods (corresponding to “This work” in Tables 2–4) is shown.

**Figure 5:**
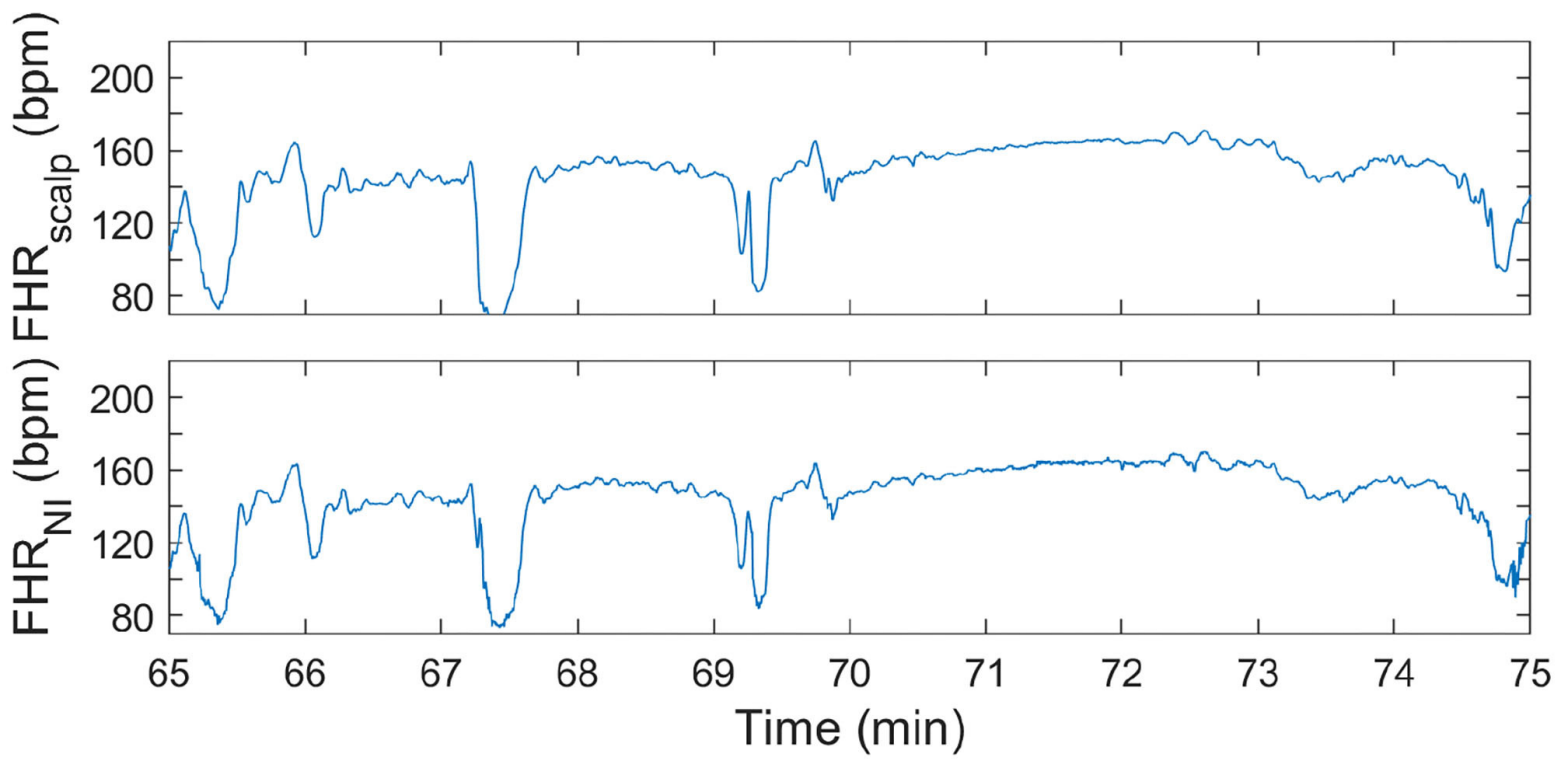
FHR tracing during second stage of labor. In the **(top)** panel, the FHR from FSE is depicted. In the **(bottom)** panel, the FHR determined from the non-invasive fetal ECG (FHR_NI_) with the proposed methods (corresponding to “This work” in Tables 2–4) is shown.

In Figures 4, 5, two examples of FHR tracings that were obtained with the presented method are shown relative to the FHR that was simultaneously obtained with a FSE. These two examples are from different patients and show FHR during the first and second stage of labor, respectively.

It can be seen in both these Figures that the resemblance between FHR patterns obtained with the presented method is high compared to the FHR patterns obtained with FSE. When looking in more detail, in Figure 5 it can be seen that the FHR deceleration at 67.5 min is slightly underestimated by the presented method. While the FSE reveals a drop in FHR to 65 beats-per-minute (bpm), the presented methods shows a drop to 75 bpm. Despite this difference, the depicted FHR patterns can be considered to be clinically equivalent.

In Tables 2–4, the results of the quantitative comparisons between the developed method and the reference methods are provided. In Table 2, the overall results are provided, while in Tables 3, 4 the results are divided in first and second stage of labor, respectively.

**Table 2:** Performance of various methods for FHR detection, as compared to the FHR from FSE as ground truth.

	**Overall**				
**Metric**	**This work**	**This work**	**NI-fECG**	**Monica AN24**	**Ultrasound**
	**(validation)**	**(train)**			
Success rate (%)	99.9 ± 0.2	99.6 ± 3.4	89.5 ± 10.8	83.4 ± 20.1	82.5 ± 21.1
CI (%)	99.9–100.0	99.5–99.6	87.9–91.1	78.8–87.9	77.8–87.3
Reliability (%)	95.7 ± 4.3	95.7 ± 6.2	86.8^*^ ± 16.3	81.7 ± 20.5	73.0 ± 24.6
CI (%)	95.3–96.0	95.6–95.8	84.2–89.5^*^	77.1–86.4	67.4–78.5
Accuracy (bpm)	3.2 ± 1.4	3.0 ± 0.6	−1.5^**^ ± 4.2	5.3 ± 2.4	10.9 ± 5.8
CI (bpm)	3.1–3.3	2.9–3.0	−3.4–0.5	4.7–5.8	9.6–12.2
LoA (bpm)	−9.2–15.5	−9.1–15.0	−29.2–26.3	Not provided	Not provided

*The results are aggregated over all patients and entire recordings. Confidence intervals for success rate and reliability are truncated at 100.0% and confidence intervals for accuracy are truncated at 0.0 (except for NI-fECG due to the different accuracy metric used)*.

* *In the paper by (12) on NI-fECG a margin of 10 bpm difference with FHR from FSE was used, instead of a margin of 10%, to assess reliability*.

** *In the paper by (12) on NI-fECG the accuracy is determined as the average of the signed differences between the FHR from NI-fECG and the FHR from FSE instead of absolute differences. CI, 95% confidence interval; LoA, limits of agreement*.

**Table 3:** Performance of various methods for FHR detection, as compared to the FHR from FSE as ground truth.

	**Stage 1**				
**Metric**	**This work**	**This work**	**NI-fECG**	**Monica AN24**	**Ultrasound**
	**(validation)**	**(train)**			
Success rate	99.9 ± 0.2	99.5 ± 3.7	91.3 ± 9.9	86.4 ± 21.1	82.6 ± 24.4
CI	99.5–100.0	92.1–100.0	89.8–92.8	81.6–91.2	77.0–88.2
Reliability	96.0 ± 3.4	95.7 ± 6.4	88.4^*^ ± 14.6	84.9 ± 21.5	74.7 ± 28.2
CI	88.8–100.0	83.0–100.0	86.0–90.8^*^	80.0–89.8	68.2–81.2
Accuracy (bpm)	3.0 ± 1.8	2.9 ± 0.6	−1.4^**^ ± 3.7	4.5 ± 2.4	7.9 ± 4.2
CI (bpm)	0.0–6.8	1.7–4.0	−3.2–0.4	3.9–5.0	7.4–10.0
LoA (bpm)	−8.3–14.3	−8.5–14.3	−27.2–24.4	−8.7–8.4	−28.4–22.7

*The results are aggregated over all patients and for first stage of labor of the recordings. Confidence intervals for success rate and reliability are truncated at 100.0% and confidence intervals for accuracy are truncated at 0.0 (except for NI-fECG due to the different accuracy metric used)*.

* *In the paper by (12) on NI-fECG a margin of 10 bpm difference with FHR from FSE was used, instead of a margin of 10%, to assess reliability*.

** *In the paper by (12) on NI-fECG the accuracy is determined as the average of the signed differences between the FHR from NI-fECG and the FHR from FSE instead of absolute differences. CI, 95% confidence interval; LoA, limits of agreement*.

**Table 4:** Performance of various methods for FHR detection, as compared to the FHR from FSE as ground truth.

	**Stage 2**				
**Metric**	**This work**	**This work**	**NI-fECG**	**Monica AN24**	**Ultrasound**
	**(validation)**	**(train)**			
Success rate	99.9 ± 0.1	99.8 ± 0.2	63.3 ± 21.7	75.2 ± 19.2	77.8 ± 21.1
CI	99.5–100.0	99.4–100.0	58.7–67.8	69.4–81.1	71.4–84.1
Reliability	85.9 ± 8.6	81.5 ± 11.4	68.5^*^ ± 24.5	71.9 ± 20.4	61.7 ± 24.8
CI	58.5–100.0	58.1–100.0	62.9–74.1^*^	65.7–78.1	54.2–69.2
Accuracy (bpm)	6.6 ± 6.3	9.4 ± 2.7	−1.7^**^ ± 8.2	7.9 ± 4.2	16.1 ± 7.6
CI (bpm)	0.0–26.6	4.0–14.9	−5.4–2.0	6.6–9.2	13.8–18.5
LoA (bpm)	−11.4–24.6	−19.1–37.9	−42.4–39.0	−12.3–12.4	−40.9–34.0

*The results are aggregated over all patients and second stage of labor of the recordings. Confidence intervals for success rate and reliability are truncated at 100.0% and confidence intervals for accuracy are truncated at 0.0 (except for NI-fECG due to the different accuracy metric used)*.

* *In the paper by (12) on NI-fECG a margin of 10 bpm difference with FHR from FSE was used, instead of a margin of 10%, to assess reliability*.

** *In the paper by (12) on NI-fECG the accuracy is determined as the average of the signed differences between the FHR from NI-fECG and the FHR from FSE instead of absolute differences*.

*CI, 95% confidence interval; LoA, limits of agreement*.

As mentioned before, the tables do not only provide the results of the presented method on the validation set but also on the train set, to enable the assessment of potential overfitting of the AI to the train set. When comparing the results for the train and validation set, it can be argued that these are comparable and hence the risk that the results are indeed overfitted is small. In fact, the performance on the validation set might be even slightly better than that on the train set.

## 4. Discussion

In this paper a new modular methodology for non-invasive electrophysiology for FHR acquisition was described. The method consists of various modules that have been individually developed and published, but with the ultimate goal of reliable FHR monitoring in mind. In the current paper, the mutual dependencies of the modules are described and an improved module for FHR detection was described that, based on the results, makes a relatively large difference in performance.

For the comparison between the presented method and other non-invasive FHR methods, the discussion below focuses on the results that are presented in the column “This work (validation)” in Tables 2–4. In this comparison, the presented method shows overall, and during the first stage of labor, significantly higher success rate and reliability, with an accuracy that is comparable to other electrophysiology-based methods and that is better than that of Doppler ultrasound. It should be noted here that the quantitative measure for accuracy is chosen according to the literature (6, 12) and defined such that the lower the value, the better the accuracy.

During the second stage of labor, success rate is significantly higher, reliability is more than 10% higher than that of Monica AN24, but the accuracy is only slightly better than that of Monica AN24 and of the previous version of the Nemo Healthcare product (i.e., NI-fECG in Tables 2–4). The main reason for this relatively smaller yield in accuracy is that the presented method has a success rate of close to 100%. During strong contractions with active pushing of the mother, the signal quality of the electrophysiological data is reduced significantly and the chance of providing inaccurate results is therefore higher. Moreover, during these episodes the FHR typically decelerates to <100 bpm. As can be seen in Figure 5, the proposed method also shows these decelerations but they are typically slightly less pronounced, yielding the same clinical picture, but at the same time producing difference between FHRs that are in the range of 10–15 bpm. In comparison, the Monica AN24 and the previous version of the Nemo Healthcare monitor show lower success rates which in practice means that during these decelerations, when the signal quality is lower, they do not show FHR, ensuring that inaccurate FHR during these episodes does not lead to erroneous interpretation but also that these inaccurate FHRs do not accumulate in a even further reduced accuracy.

### 4.1. Limitations

This study has four main limitations. First, in this study we have followed a common approach in evaluating the performance of AI methods by using a holdout dataset for validation. This holdout set can demonstrate the generalizability of the trained AI. In this study, however, the size of the validation set is relatively small. To still provide some insights in the potential of the proposed method on a larger dataset, we have included the results on the training data in Tables 2–4. While these results might be overestimating the performance of the proposed method due to overfitting, it should be noted here that the chance of overfitting is small. Not only are the results of the validation set similar to those of the training set, but also are the results of the AI not used directly to determine the FHR. More specifically, the results of the AI are used in the *prior* model of the hierarchical R-peak detection method and additional models—that e.g., consider the morphology of the signal at the expected position of the R-peak—are employed that likely prevent the detection of overfitted FHR values.

A second limitation of the study is that the comparison to other methods is indirect. The various methods have each been evaluated on their own data sets, with different numbers of patients, different patient characteristics, and different lengths of recordings. Therefore, no quantitative comparison can be made and no strong conclusions can be drawn about the performances of all methods. Yet, in our opinion the number of recordings in each study and the difference in performances is large enough to argue that the presented method outperforms the other methods in FHR detection during all stages of labor.

A third limitation is that the presented method, but also all reference methods, provide a FHR at 4 Hz sampling intervals. Fetal electrophysiological recordings potentially enable the study of beat-to-beat variability in the FHR which has been reported to yield better performance in detecting fetuses in distress when using linear features of fetal heart variability (3, 29). On the other hand, data resampled to 4 Hz has been reported to yield similar effects on features that reveal physiological changes during progression of labor and even better performance on detecting fetuses in distress when using entropy features of heart rate variability (29). Moreover, the communication protocols for most central monitoring systems require FHR values to be communicated at fixed frequency of 4 Hz. Because of the absence of a clearly best method to communicate FHR values (i.e., on a beat-to-beat basis or at a fixed frequency) and to adhere to the existing communication protocols and bring the methods presented in this paper already one step closer to implementation in clinical practice, we have chosen to equidistantly resample our data to 4 Hz.

The fourth limitation of the study is that all data processing presented in this study was done offline, on a desktop computer. While the non-AI parts of the method can be processed online (i.e., processing e.g., 1 s of data takes <1 s) on a normal desktop computer, the AI extension takes on average 2 s to process 1 s of data on a GPU (Titan V, NVIDIA, USA), when implemented in Tensorflow-Keras. Related to this limitation, unlike the reference methods shown in Tables 2–4, the presented method is not yet implemented in a medical device. Efforts to achieve this are currently ongoing.

### 4.2. Future Potential

Other than the implementation of the presented methods in a clinical device for reliable and unobtrusive FHR monitoring, the presented methods might have further potential to support obstetrical healthcare. Because the transabdominal recordings can, with relatively small additional effort, also provide the fetal ECG (30), further analysis of the ECG morphology, such as ST analysis might be possible. For ST analysis, accurate normalization of the fetal orientation would be crucial however. Fetuses in cephalic, transverse, or breech position would give different ECG morphology. We have shown in a previous study (31) that a different fetal orientation, or a different orientation of the electrical heart axis with respect to the abdominal electrodes, affects the degree of ST elevation and as such might affect ST alarms triggered on an obstetrical ward. Normalization for the fetal orientation would be possible by using ultrasound imaging (30) or different (i.e., relative) ST alarm mechanisms (32).

## 5. Conclusions

In this paper, a new method for FHR detection from non-invasive, transabdominal electrophysiological measurements was presented. The method is able to determine a reliable FHR in >95% of time during labor, making it substantially more reliable and accurate than Doppler ultrasound—the current clinical standard for non-invasive cardiotocography. During second stage of labor, the performance of the method decreases, but with a reliability higher than 80% it still outperforms Doppler ultrasound and other reference methods by a significant amount.

## Data Availability Statement

The data analyzed in this study is subject to the following licenses/restrictions: Data are available from the Data Governance Board of the Máxima Medical Center for researchers who can demonstrate they are qualified to use confidential data. Requests to access these datasets should be directed to Rik Vullings, r.vullings@tue.nl, Judith O. E. H. van Laar, judith.van.laar@mmc.nl.

## Ethics Statement

The studies involving human participants were reviewed and approved by Máxima Medical Center institutional review board. The patients/participants provided their written informed consent to participate in this study.

## Author Contributions

RV was responsible for data processing and analysis, statistical analysis, and drafting of the manuscript. JL was responsible for data collection and reviewing of the manuscript. All authors contributed to the article and approved the submitted version.

## Conflict of Interest

RV is co-founder and share holder in Nemo Healthcare BV, the Netherlands. The remaining author declares that the research was conducted in the absence of any commercial or financial relationships that could be construed as a potential conflict of interest.
